# An MSI Tumor Specific Frameshift Mutation in a Coding Microsatellite of MSH3 Encodes for HLA-A0201-Restricted CD8^+^ Cytotoxic T Cell Epitopes

**DOI:** 10.1371/journal.pone.0026517

**Published:** 2011-11-14

**Authors:** Yvette Garbe, Claudia Maletzki, Michael Linnebacher

**Affiliations:** 1 National Center for Radiation Research in Oncology, Dresden, Germany; 2 Division of Molecular Oncology and Immunotherapy, Department of General Surgery, University of Rostock, Rostock, Germany; International Centre for Genetic Engineering and Biotechnology, Italy

## Abstract

**Background:**

Microsatellite instability (MSI) resulting from inactivation of the DNA mismatch repair system (MMR) characterizes a highly immunological subtype of colorectal carcinomas. Those tumors express multiple frameshift-mutated proteins which present a unique pool of tumor-specific antigens. The DNA MMR protein MSH3 is frequently mutated in MSI^+^ colorectal tumors, thus making it an attractive candidate for T cell-based immunotherapies.

**Methodology/Principal Findings:**

FSP-specific CD8^+^ T cells were generated from a healthy donor using reverse immunology. Those T cells specifically recognized T2 cells sensitized with the respective peptides. Specific recognition and killing of MSI^+^ colorectal carcinoma cells harbouring the mutated reading frame was observed. The results obtained with T cell bulk cultures could be reproduced with T cell clones obtained from the same cultures. Blocking experiments (using antibodies and cold target inhibition) confirmed peptide as well as HLA-A0201-specificity.

**Conclusions:**

We identified two novel HLA-A0201-restricted cytotoxic T cell epitopes derived from a (-1) frameshift mutation of a coding A(8) tract within the MSH3 gene. These were ^386^-FLLALWECSL (FSP18) and ^387^-LLALWECSL (FSP19) as well as ^403^-IVSRTLLLV (FSP23) and ^402^-LIVSRTLLLV (FSP31), respectively. These results suggest that MSH3(-1) represents another promising MSI^+^-induced target antigen. By identifying two distinct epitopes within MSH3(-1), the sustained immunogenicity of the frameshift mutated sequence was confirmed. Our data therefore encourage further exploitation of MSH3 as a piece for peptide-based vaccines either for therapeutic or –even more important– preventive purposes.

## Introduction

Loss of the DNA mismatch repair (MMR) system by (epi-) genetic alterations leads to an increased mutation rate in short, tandemly repeated sequences, termed microsatellites. This phenomenon, commonly referred to as microsatellite instability (MSI), is presented by length variations in tracts of mono- or polynucleotides.

Clinically, MSI is found in >90% of patients affected by the hereditary non-polyposis colorectal carcinoma (HNPCC) syndrome, as well as in several sporadic malignancies including tumors of the colorectum, the stomach and the endometrium, where it is found in up to 15% of cases. When comparing with microsatellite stable tumors, there is some evidence for –at least partial– immunological growth control in MSI cancers, like (I) the dense local lymphocytic infiltration (“Crohn's-like lymphocytic reaction”), (II) the increased apoptotic tumor cell number, and (III) the low number of distant metastases that (IV) leads to an improved overall patient survival [1]–[3]. Beyond that, there is evidence that MMR deficient cells are intrinsically resistant to methylating agents and to some antimetabolites, including the chemotherapeutic drug 5-Fluorouracil, which is standard in adjuvant treatment of colorectal carcinoma (CRC) [4].

In the multistep process of carcinogenesis, mutations affecting genes, whose alterations are advantageous to the tumor cell, will be positively selected. In MSI^+^ cancers, several genes being especially prone to MSI have been identified with the transforming growth factor beta receptor II (TGFβRII) being one of the first. Other examples of so-called MSI target genes frequently mutated in CRC include Caspase-5, ACVR2, and AIM2 [5], [6].

From a biochemical point of view, MSI affecting coding regions of genes leads to frameshift mutations and the synthesis of C-terminally modified proteins. The resulting altered proteins typically lack normal functionality but additionally, they constitute neo-epitopes, when presented in the context of MHC molecules at the tumor cells' surface. In recent years, our group was leading in demonstrating the high immunogenicity of MSI-induced frameshift-peptides (FSP) by identifying numerous epitopes recognized by T cells [7]–[10]. Using the classical reverse-immunology approach, T cells from healthy HLA-A0201^+^ donors are stimulated by synthetic FSPs. Importantly, the outgrowing T cells are mainly CD8^+^ cytotoxic T lymphocytes (CTL) capable to effectively lyse cells harbouring the respective mutation [7], [10], [11], [12]. Based on these *in vitro* studies, Schwitalle et al. provided evidence for FSP-specific immune responses not only in HNPCC patients but also in still healthy HNPCC germline mutation carriers [13]. This study additionally revealed that FSPs are recognized by the human immune system and thus represent relevant tumor antigens *in vivo*.

Hence, FSPs are very interesting targets for specific immunological approaches both for therapeutic and –even if currently hypothetical–for preventive purposes. Since an effective tumor vaccine ideally contains epitopes derived from several tumor-specific antigens, the identification of additional, relevant T cell epitopes is imperative.

Here, we describe the identification of HLA-A0201-restricted CTL epitopes generated by a MSI^+^ tumor specific frameshift mutation in a cMS of the MSH3 gene. MSH3 is one of the DNA MMR genes located on chromosome 5q14.1. It belongs to the MutS family and mutations are found in about 40% of colorectal and stomach cancers, as well as in >50% of established CRC cell cultures [14]. Functional loss of this protein was found to play a role in the progression of MMR-deficient tumors by increasing instability [15]. Therefore, mutated MSH3 shows several characteristics an ideal tumor antigen should have and thus constitutes another important candidate for the development of immune-based MSI^+^ tumor targeting strategies.

## Materials and Methods

### Peptides

HLA-A0201-restricted peptides against the (-1) mutation of a A(8) tract within the MSH3 gene were selected on the basis of two peptide-motif scoring systems as described before [9]. Using these methods, 12 peptides were selected as candidate T cell epitopes including six 9-mer, and six 10-mer peptides. Peptides were purchased from the Peptide Synthesis Facility of the German Cancer Research Center, dissolved in DMSO (5 mg/ml) and further diluted in PBS (500 µg/ml). A complete list of peptides used in this study is provided in Table 1.

**Table 1:** Frameshift and control peptides used in this study.

Protein	Accession number^a^	Name	Peptide^b^	Theoretical Scores^c^		Fluorescence Index^d^
				Ken Parker SYFPEITHI		
MSH3(-1)	AAB47281	FSP17	^389^-ALWECSLPQA	389	24	4.42
MSH3(-1)	AAB47281	FSP18	^386^-FLLALWECSL	364	25	4.85
MSH3(-1)	AAB47281	FSP19	^387^-LLALWECSL	36	26	1.16
MSH3(-1)	AAB47281	FSP20	^394^-SLPQARLCL	21	23	0.05
MSH3(-1)	AAB47281	FSP21	^402^-LIVSRTLLL	5	23	0.01
MSH3(-1)	AAB47281	FSP22	^401^-CLIVSRTLL	21	22	0.2
MSH3(-1)	AAB47281	FSP23	^403^-IVSRTLLLV	24	21	0.13
MSH3(-1)	AAB47281	FSP24	^382^-KRATFLLAL	0.1	20	0.01
MSH3(-1)	AAB47281	FSP31	^402^-LIVSRTLLLV	37	25	0.2
MSH3(-1)	AAB47281	FSP32	^394^-SLPQARLCLI	24	24	0.01
MSH3(-1)	AAB47281	FSP33	^401^-CLIVSRTLLL	21	23	1.98
MSH3(-1)	AAB47281	FSP34	^399^-RLCLIVSRTL	4	22	0.34

a Protein or nucleotide accession numbers are indicated;

b Position of the start amino acid in the protein is indicated;

c Predicted binding scores to HLA-A0201 using computer-assisted analysis;

d (mean fluorescence with peptide - mean fluorescence without peptide)/(mean fluorescence without peptide).

### Cell lines

All tumor cell lines were obtained from the German Collection of Microorganisms and Cell Cultures (Braunschweig, Germany) or from Cell Lines Services (Eppelheim, Germany) tumor bank and grown in RPMI 1640 medium supplemented with 10% fetal calf serum (FCS) and 2 mmol/L L-glutamine. The MSI^+^ colorectal carcinoma cell lines HCT116 [HLA-A0201^+^; MSH3(-1)], and Colo60H [HLA-A0201^+^; MSH3(-1)], as well as HLA-A0201^+^ T2 cells, exogenously loaded with peptide, were used for functional analysis. As controls, the MSS colorectal carcinoma cell lines SW480 and SW707 [both HLA-A0201^+^] as well as the erythroleukemic HLA^−^ cell line K562 were applied. All tissue culture media and supplements were purchased from PAA (Cölbe, Germany) unless indicated otherwise. IL-4, IL-7, and IFN-γ were obtained from Cellgenix (Freiburg, Germany); Proleukine (IL-2) was obtained from Novartis (Nürnberg, Germany). The cells were maintained in a humidified 37°C incubator with 5% CO_2_.

### Generation of CD40-activated B cells & Peptide-specific T cell stimulation

All procedures were performed as described before [10]. Briefly, peripheral blood mononuclear cells (PBMC) were isolated from a buffy coat of a healthy HLA-A0201^+^ donor by ficoll-density gradient centrifugation. The use of buffy coats for scientific investigations has been approved by the institutional review board of the university of Heidelberg.

Thereafter, T cells were purified by magnetic depletion of non T cells using the Pan T cell isolation kit II (Miltenyi Biotec, Bergisch-Gladbach, Germany) according to the manufacturer's instructions.

CD40-activated B cells (CD40Bs) were generated from the same healthy HLA-A0201^+^ donor via NIH/3T3 feeder cells, stably transfected with human CD154. Outgrowing CD40Bs were restimulated every 3–4 days. CD40 Bs were irradiated (30 Gy), incubated with peptide (10 µg/ml; peptides were applied as mixes of four peptides each) in serum-free IMDM for 1 hour at 37°C, and washed to remove excess of peptide. Then the CD40 Bs were added to the purified autologous T cells at a ratio of (T:CD40 Bs) 4∶1 in T cell medium (IMDM containing 10% FCS, 2 mmol/l L-glutamine, and antibiotics), supplements (1∶100), and IL-7 (10 IU/ml). T cells were restimulated every 7 days; IL-7 was replaced by IL-2 (100 IU/ml) from day 28 on. T cell cultures were repeatedly characterized by flow cytometry following extracellular and intracellular staining protocols as described [9].

### Cloning of FSP-specific T cells by limiting dilution from T cell bulk cultures

Cloning of FSP18, 19, 23, and 31-specific cytotoxic T lymphocytes (CTL) was performed by limiting dilution of the T cell bulk culture. Hence, T cells (0.7 cells/well) were added to V-bottomed 96-well plates containing 3×10^5^ lethally irradiated (30 Gy) peptide-loaded autologous CD40Bs at a final volume of 200 µl IMDM (supplemented with 10% FCS, supplements (1∶100), and IL-2 (100 IU/ml)). This treatment was repeated weekly for restimulation of T cells. After 4–6 weeks, specificity of outgrowing T cells was tested in IFN-γ-ELISpot (Enzyme-linked immunospot) assays.

### ELISpot assays

ELISpot assays were accomplished using nitrocellulose-lined 96-well-plates (Multiscreen, Millipore, Bedford, MA, USA) covered with mouse anti-human IFN-γ mAb (Mabtech, Nacha, Sweden) and blocked with serum-containing medium. T cells (1×10^4^ cells/well) were then added to each well along with T2 target cells (3.5×10^4^ cells/well), which had been preincubated with cognate peptides (1.5 µg/well) or with irrelevant control peptide (^128^-YLLPAIVHI from P68) as a negative control. Following 16 hours incubation in a 5% CO_2_ humidified chamber at 37°C, the wells were washed vigorously six times with PBS-Tween (PBS+0.05% Tween-20) and incubated with a biotinylated rabbit anti-human IFN-γ mAb for 4 h, washed again, incubated with streptavidin–alkaline phosphatase for 2 h, and washed again. Spots were visualized by incubation with BCIP/NBT (Sigma-Aldrich, Steinheim, Germany) for up to 1 h. The reaction was stopped with tap water, and after drying, the spots were counted using the KS-ELISpot reader (Zeiss Kontron, Göttingen, Germany).

To confirm HLA-A2^+^ restriction of FSP-specific CTLs, antibody-blocking ELISpots were performed. FSP-loaded T2 were incubated with the monoclonal antibodies anti-HLA-A2 (clone BB7.2) and pan-anti-MHCI (clone W6/32) and T cells with anti-CD3 (clone OKT3) and anti-CD8 (clone OKT8).

### Chromium release assay

Standard chromium release assays were performed as described [9]. The percentage of specific lysis was calculated as follows:
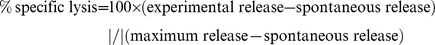



## Results

### Induction of highly activated cytotoxic T cells specific for MSH3(-1)-derived peptides

FSP-specific T cell bulk cultures were generated from a healthy HLA-A0201^+^ donor using mixes of hypothetically immunogenic peptides, generated by a (-1) frame of the MSH3 gene. In order not to miss a potential T cell epitope, different peptides, including six 9-mers and six 10-mers, were tested (see Table 1 for details). For initial T cell stimulation experiments, peptide pools, consisting each of four peptide sequences, were applied. Autologous CD40Bs were employed as antigen presenting cells. These CD40Bs were loaded with peptide mixes and used for weekly restimulation of T cells.

With this method, three different FSP-specific T cell bulk cultures were successfully established. Following seven to eight weeks of restimulation, T cell numbers increased >100-fold in each culture (data not shown). Detailed flow cytometric characterization of proliferating T cell cultures revealed generation of highly pure and activated T cells. As can be depicted from Table 2, cell surface as well as intracellular staining of T cells stimulated against FSPs 31–34 identified that the bulk culture consisted primarily of CD8^+^ cytotoxic T cells at day 35. Further culture resulted in a nearly exclusive cytotoxic phenotype (CD8^+^: 97.1% at day 98 of culture). Most of these T cells expressed high levels of the activation markers CD25 (98.1%) and CD71 (99.4%). Intracellular staining showed that a significant part of these cytotoxic T cells were positive for TNF-α, IFN-γ, and granzyme B, while the minority expressed IL2 and perforin (Table 2). Similar results were obtained for the two other T cell bulk cultures generated against FSP 17–20 and FSP 21–24 (Table 2).

**Table 2:** FACS analysis of the T cell bulk culture.

Antigen	Tc bulk culture (% positive cells)		
	Tc FSP17–20	Tc FSP21–25	Tc FSP31–34
CD2^1^	91.7	98.6	99.4
CD3^1^	99.5	99.7	99.5
CD4^1/3^	22.4/1.7	35.8/6.7	28.1/5.0
CD8^1/3^	71.0/97.1	68.9/91.5	67.8/94.9
CD28^1^	64.8	84.4	79.5
CD45R0^1^	66.3	94.7	94.5
CD49d^1^	80.7	88.0	79.5
CD50^1^	99	99.7	99.6
CD25^1/3^	16.8/94.1	38.3/96.9	36.2/98.1
CD69^1/3^	35.5/43.9	13.9/73.5	36.4/45.7
CD71^3^	99.5	99.2	91.6
IFN-γ^2^	68.6	73.6	42.9
TNF-α^2^	ND	ND	66.9
IL-2^2^	ND	ND	22.5
Perforin^2^	11.6	13.2	11.1
Granzyme B^2^	96.1	92.5	97.5

1 d35 of Tc culture, extracellular staining;

2 d86 of Tc culture, intracellular staining;

3 d98 of Tc culture, extracellular staining; ND - not done.

### Recognition and killing of peptide-loaded T2 cells by T cell bulk cultures

Next, peptide recognition and target cell killing of the expanded T cell bulk cultures was determined. FSP-specificity was examined using T2 cells exogenously loaded with the respective peptides. T2 cells loaded with irrelevant peptide (P68) served as negative control.

**Figure 1:**
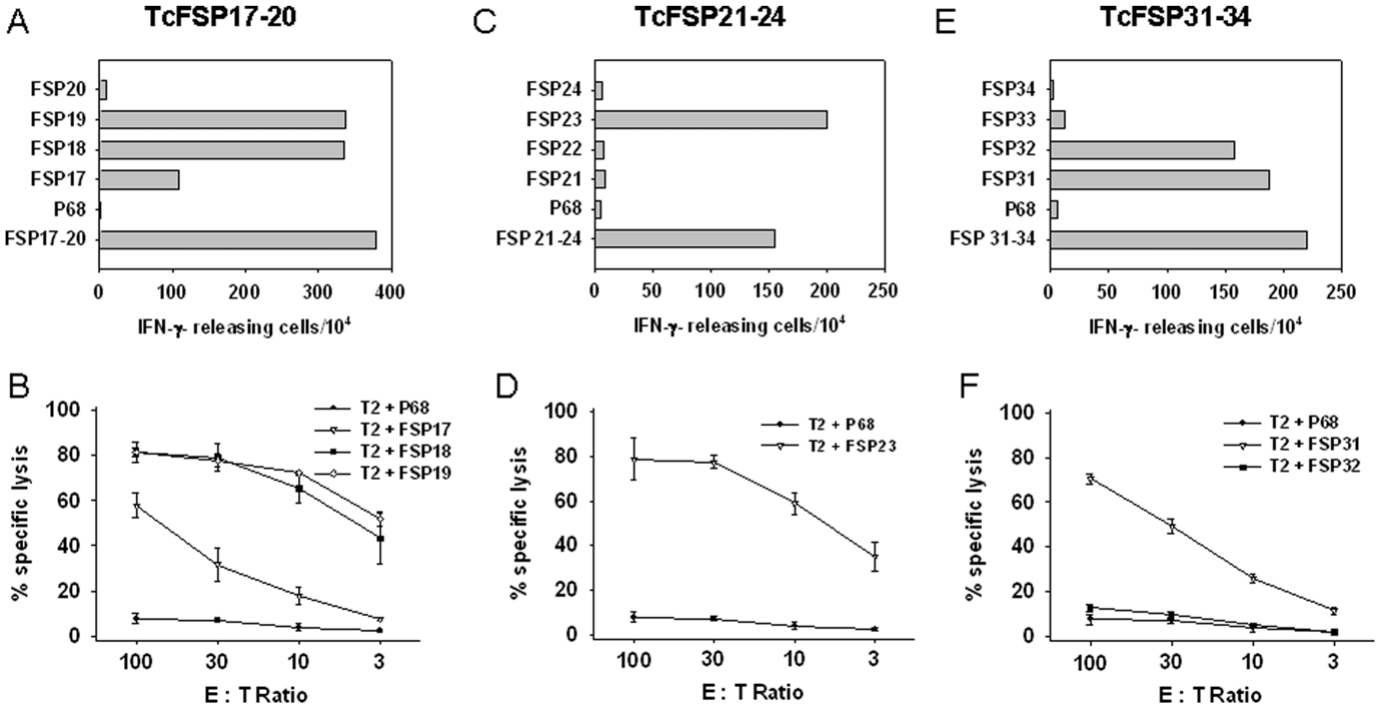
IFN-γ ELISpot and ^51^Cr-release assays. FSP-specific T cells were stimulated against MSH3(-1) derived peptide mixes FSP17–20, FSP21–24, and FSP31–34. Thereafter, outgrowing T cell cultures were incubated for 5 h with peptide-loaded T2 cells as described in “material and methods”. (A, C and E) show the number of IFN-γ releasing T cells (B, D and F) show cytotoxic activity of FSP-specific T cells. Results are displayed as the mean and standard deviation from analysis performed in triplicate.

As can be depicted from Figure 1A, T cells that had been raised against a mixture of FSP17–20 exhibited a very strong response towards ^386^-FLLALWECSL (FSP18) and ^387^-LLALWECSL (FSP19). The number of IFN-γ secreting T cells was comparable to those stimulated with the peptide mix, indicative for specific recognition of these peptide sequences. Both peptides cover the same epitope with FSP18 being a 10mer peptide and FSP19 a 9mer peptide. Thereafter, specific killing activity of these T cells was investigated. They effectively lysed T2 cells loaded with both FSP18 and 19 (Figure 1B). Interestingly, minor T cell reactivity was observed towards FSP17 in ELISpot assays and accordingly lysis of FSP17-loaded T2 cells was less efficient (Figure 1B). Contrary, FSP17–20 bulk T cells displayed no recognition of FSP20 and this peptide was not analyzed further.

Testing of the CTLs stimulated against FSP21–24 revealed an exclusive recognition of the peptide ^403^-IVSRTLLLV (FSP23), while reactivity was completely absent towards the other peptides present in this mix (Figure 1C). In a subsequent cytotoxicity assay, T cells exhibited pronounced lytic potential towards FSP23-loaded T2 cells (Figure 1D).

Analysis of the FSP31–34 T cell bulk culture revealed a strong recognition of ^402^-LIVSRTLLLV (FSP31) and ^394^-SLPQARLCLI (FSP32) in IFN-γ-ELISpot assays. Approximately 1.5 and 1.9% (FSP31 and 32, respectively) of the T cells specifically secreted IFN-γ in response to peptide loaded T2 targets (Figure 1E). However, cytolytic activity was observed exclusively towards FSP31-loaded T2 cells. FSP32 did not elicit a reaction (Figure 1F). Moreover, there was no detectable recognition of FSP33 and 34.

Taken together, these data provide clear evidence for the generation of T cell cultures specific for several epitopes generated by the MSI-induced (-1) frameshift mutation of MSH3.

### Reactivity towards MSI^+^ colorectal tumor cells

**Figure 2:**
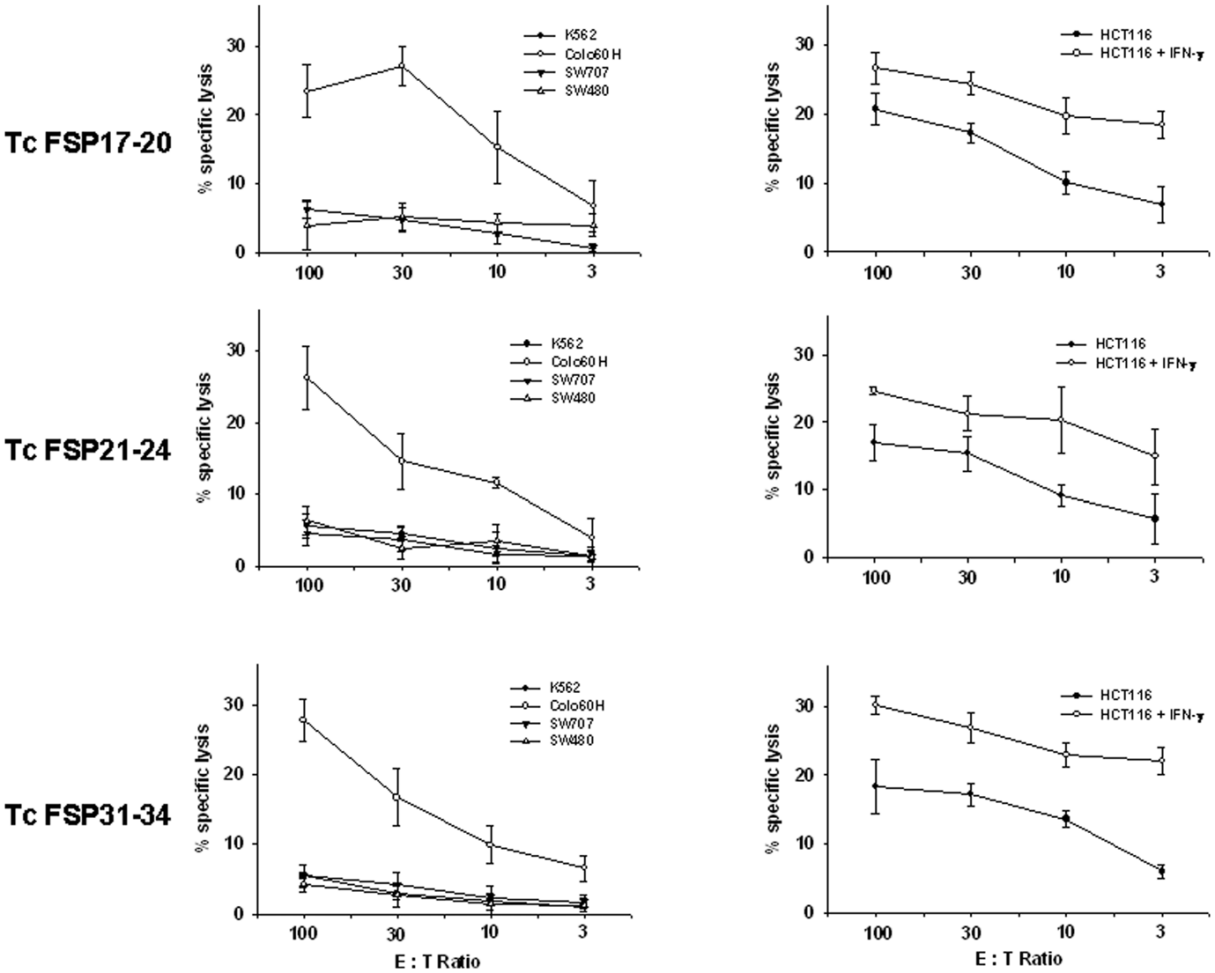
Functional recognition of target cells by FSP specific T cells. Specific lysis of HLA-A0201^+^ cell lines harbouring a (−1) version of MSH3 (left panel). Specificity was confirmed by lack of reactivity against the colorectal carcinoma cell lines SW480 and SW707 (HLA-A0201^+^, MSH3 (wt)). Lytic potential was slightly enhanced by pre-treatment of HCT116 cells with IFN-γ for 24 h (left panel). T,arget cell lysis is depicted at different effector to target cell (E∶T) ratios. All results are displayed as the mean and standard deviation from analysis performed in triplicate.

To analyze, if the observed reactivity against MSH3(-1)-derived peptides was confined to peptide-loaded target cells, killing activity towards MSI^+^ tumor cells was examined. FSP17–20 CTL bulk cultures specifically recognized the MSI^+^ CRC cell lines HCT116 and Colo60H, which express a (-1) version of MSH3 endogenously (Figure 2A, left panel). Susceptibility of HCT116 towards T cell mediated killing was slightly enhanced by IFN-γ pre-treatment (Figure 2A, right panel) which leads to an upregulation of MHC-I and HLA-A0201 on this tumor cell line [9]. Comparable results were obtained for T cells raised against FSP21–24 and FSP31–34. As can be depicted from Figure 2B and C, T cells effectively eliminated target cells harbouring the underlying MSH3(-1) mutation. Again, killing potential was increased by overnight treatment of HCT116 cells with IFN-γ (Figure 2B and C, right panel).

This reactivity was likely to be specific since none of the CTL bulk cultures reacted towards the HLA-A0201^+^ but microsatellite stable cell lines SW480 and SW707. Moreover, unspecific NK cell-mediated killing could be excluded by lacking reactivity towards the classical NK cell target K-562 (Figure 2).

In summary, these results provide evidence for the recognition of several MSH3(-1) peptides presented in HLA-A0201 molecules after endogenous expression and proteasomal processing.

### Generation of FSP-specific T cell clones by limiting dilution from CTL bulk cultures

The gold standard in T cell epitope discovery is the confirmation of target cell recognition using T cell clones. Hence, we next established clones by classical limiting dilution of all three CTL bulk cultures showing strong reactivity in ELISpot and cytotoxicity assays. Precisely, this was done using autologous CD40Bs loaded with FSP18, 19, 23, and 31.

Following several weeks of restimulation, outgrowing T cell clones were harvested. Twenty-four CTL clones specific for FSP18 were obtained. In subsequent cytotoxicity assays, six of these clones were capable to lyse peptide-loaded T2 targets (Table 3). Next, the lytic activity of FSP18-specific T cell clones towards MSI^+^ tumor cells was investigated. Two clones effectively killed HCT116 cells with one clone displaying additional reactivity against Colo60H. Comparable results were obtained for FSP19. Of the 46 obtained CTL clones, FSP19-loaded T2 cell lysis was induced by 12 clones. Of those, eight were reactive towards HCT116 cells, and two clones also lysed Colo60H targets (Table 3).

**Table 3:** FSP-specific CTL clones and lysis of target cells.

			Lysis of		
Name	Peptide	Clones	T2	HCT116	Colo60H
FSP18	^386^-FLLALWECSL	24	6	2	1
FSP19	^387^-LLALWECSL	46	12	8	2
FSP23	^403^-IVSRTLLLV	20	3	2	-
FSP31	^402^-LIVSRTLLLV	66	44	18	2

A total of 66 FSP31-specific clones were established. Of note, 44 clones effectively lysed FSP31-loaded T2 cells, 18 of those clones specifically killed HCT116 targets but only two clones were highly reactive towards Colo60H cells.

Finally, from the 20 generated FSP23 clones, only three exhibited killing activity towards FSP23-loaded T2 cells. Two of these clones were capable of lysing HCT116 tumor cell targets but no reactivity was observed against Colo60H cells (Table 3).

To sum up these data, we could identify two distinct epitopes showing sustained immunogenicity out of the (-1) MSH3 frameshift mutated sequence. These are ^386^-FLLALWECSL (FSP18) and ^387^-LLALWECSL (FSP19), respectively as well as ^403^-IVSRTLLLV (FSP23) and ^402^-LIVSRTLLLV (FSP31), respectively.

### Specific HLA-A0201-restricted recognition of MSH3(-1) tumor cells by FSP31-specific T cell clones

Selected FSP31-specific CTL clones demonstrating reactivity towards tumor cell targets were further analyzed in close detail. Experiments were performed with three sufficiently growing T cell clones.

**Figure 3:**
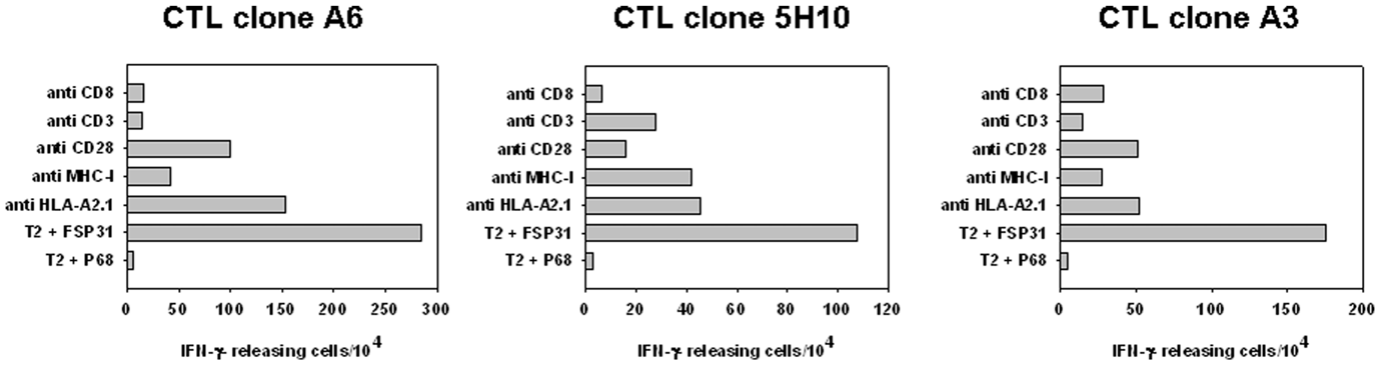
Antibody blocking ELISpot. FSP-specific CTLs were stimulated against T2 cells either loaded with FSP31 or irrelevant peptide (P68). HLA-specificity was examined by blocking MHC-I on the target cell side (HLA-A2 and pan-MHC-I). Inhibition of IFN-γ release was confirmed by blocking CD3, CD8 and CD28 on the T cell side. The number of IFN-γ releasing T cells is given. Results are displayed as the mean and standard deviation from analysis performed in triplicate.

In antibody blocking experiments, HLA-A0201 restricted target cell recognition by FSP31-specific T cell clones was confirmed. As can be taken from Figure 3, IFN-γ release by CTLs in response to FSP31-loaded T2 cells was substantially inhibited by antibodies interfering with MHC-I on the target cell side (HLA-A2 and pan-MHC-I) as well as CD3, CD8 and CD28 on the T cell side.

**Figure 4:**
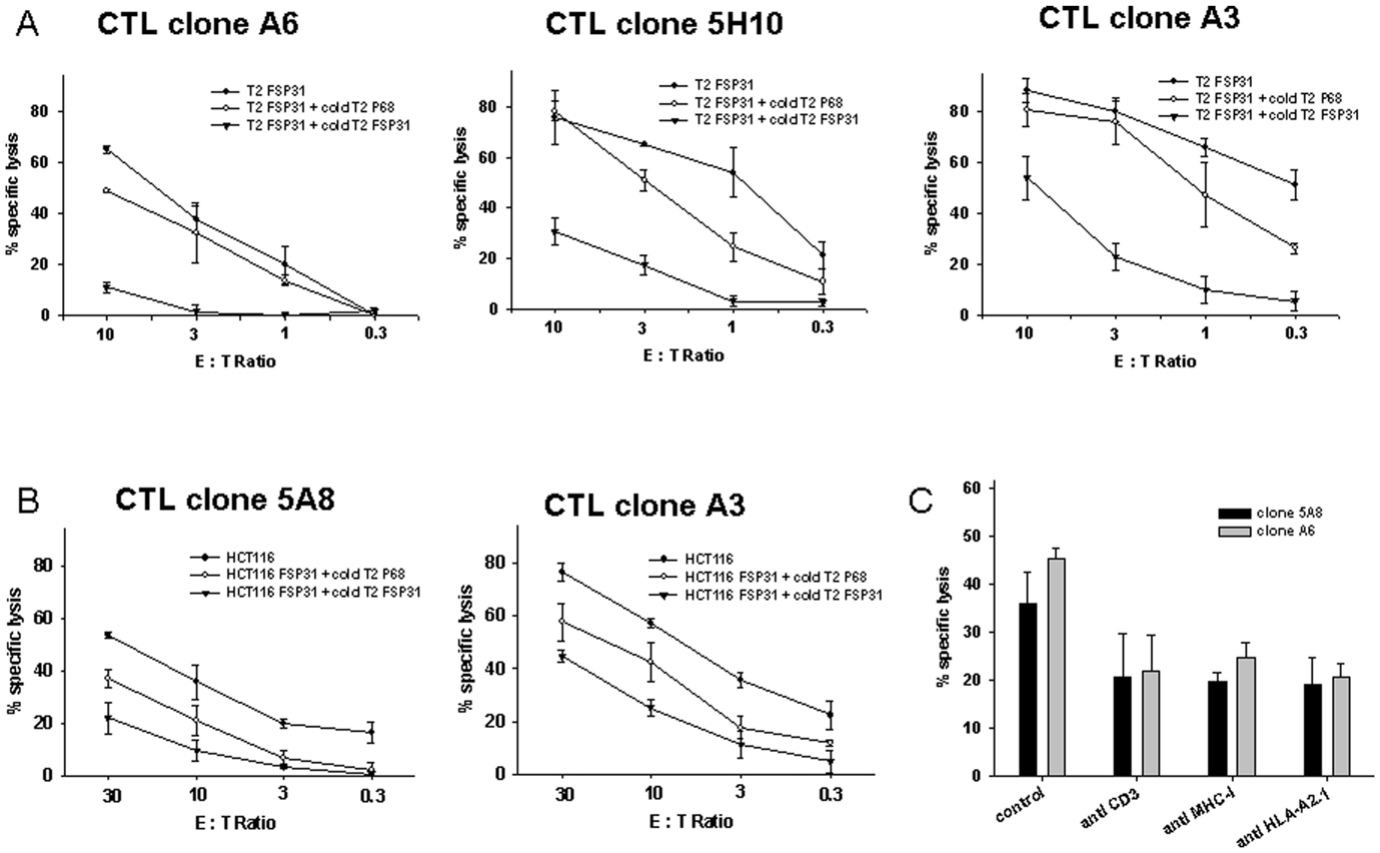
Cold target inhibition. FSP-specificity of four different CTL clones (A3, A6, 5A8 and 5H10) was examined. Firstly, an excess of unlabeled T2 cells either pulsed with FSP31 or with irrelevant peptide (P68) were added into experimental wells. Experiments were performed using (A) T2 and (B) HCT116 as target cells. Lysis of unpulsed HCT116 or exogenously FSP31-loaded T2 cells without cold targets served as control. Killing of target cells is depicted at different effector to target cell (E∶T) ratios. Secondly, recognition of HCT116 CRC target cells of two clones was blocked by addition of antibodies against CD3, HLA-A2 and pan-MHC-I at an E∶T ratio of 10∶1. All results are displayed as the mean and standard deviation from analysis performed in triplicate.

Additionally, FSP31 specificity and HLA-A0201 restriction of CTL clones could be confirmed in cold-target inhibition experiments by adding an excess of unlabeled T2 target cells into standard ^51^Cr-release assays (Figure 4). In these analysis, lysis of labeled FSP31-loaded T2 cells was substantially inhibited when an excess of unlabeled T2 cells loaded with FSP31 was present, but not when these unlabeled T2 cells were loaded with an irrelevant peptide (P68). Effects were most obvious for clones A6 and 5H10 followed by A3 (Figure 4A). In additional blocking experiments HCT116 cells were used as target cells. Tumor cell lysis of the clones was effectively inhibited by adding an excess of FSP31 loaded T2 cells. T2 cell pulsed with the irrelevant peptide P68 had only minor effect on the tumor cell killing activity (Figure 4B). Thus, this provides strong evidence for HLA-A0201 restriction and recognition of endogenous FSP31 of these CTL clones. Finally, we performed antibody blocking kill assays. Here, the reactivity against HCT116 could be blocked approximately half by antibodies against the T cell receptor CD3 and against both pan-MHC-I and HLA-A2 (Figure 4C).

## Discussion

Vaccination strategies aiming to specifically act on tumor target antigens have now come to the fore. Ideally, an efficient antitumor immunotherapy involves a multi-epitope strategy including T-cell epitopes from a set of highly immunogenic tumor-associated antigens and several different HLA-class I restriction elements. However, most of the tumor antigens identified so far may not constitute perfect target structures since antigen down-regulation or loss can occur during immunotherapy. Hence, there is a need for further identification of relevant T cell epitopes.

MSI leads to production of abnormal proteins predominantly constituting of frameshift mutants that represent neo-antigens to the immune system and thus may elicit spontaneous immune responses. Indeed, MSI^+^ tumors often exhibit marked lymphocytic infiltration, especially at the tumor invasion front in the stromal compartment, with a predominance of activated CD8^+^ T cells [16]. Consequently, it has been suggested that this may even be causative for the better prognosis observed for MSI^+^ CRC when compared to their MSS counterpart [3]. Whether the observed T cell responses are directly associated with the higher apoptosis rate of MSI^+^ tumor cells or whether this is just a coincidence is largely unsolved [16]. On the contrary, there is a correlation between the overall number of frameshift mutations and tumor-infiltrating lymphocyte density, arguing for the clinical relevance of T cell-based immunotherapies.

In the last decade, a number of MSI-induced CD4^+^ and CD8^+^ T cell epitopes derived from FSPs have been identified by us and others [7]–[12]. The relevance of these FSPs as tumor-specific antigens *in vivo* was only recently shown by providing evidence for the presence of FSP-specific immune responses not only in MSI^+^ CRC patients, but also in still healthy HNPCC germline mutation carriers [13]. This observation is a striking argument in favour of a substantial contribution to tumor growth control by FSP-specific T cells *in vivo*, making those peptides very interesting candidates for the development of targeted vaccination strategies. There are, however, still surprisingly few frameshift epitopes characterized and consequently, in order to identify the best frameshift candidates for future MSI-specific immunotherapeutic approaches, more must urgently be defined.

We here identified two epitopes derived from a (-1) frameshift mutation of a coding A(8) tract within the DNA MMR gene MSH3. These antigenic epitopes could be good candidates for immunotherapy, because mutations in the MSH3 gene, along with others, e.g. TGFβRII, BAX and MSH6, appear to play an active role in tumor progression [17]. By examining the sequence and timing of target gene alterations, it was reported that MSH3 mutations are rather late events in the multistep process of carcinogenesis, probably promoting metastasis or recurrence [18], [19]. Of note, MSH3-deficient MSI-low CRCs, corresponding with multiple tetranucleotide frameshifts, have poor clinical outcomes, indicative for driving metastasis in MSI-low CRC, too [20].

By using the strategy of reverse immunology, T cells from a HLA-A0201^+^ donor were stimulated against a pool of different peptides. Peptides were selected on algorithm-predicted candidate epitopes, hypothetically binding with high affinity to HLA-A0201 molecules thus forming stable MHC/peptide complexes. All three polyclonal bulk T cell cultures grew well and FSP-specific reactions could be observed towards half of the twelve MSH3(-1)-derived peptides included into this analysis. Of particular relevance was the finding that T cell mediated tumor cell recognition and lysis could be detected towards MSI^+^ CRC cancer cells expressing the underlying mutation endogenously. This finding is in line with our previous studies and thus extends our knowledge on MSI-derived tumor specific antigens being recognized by cytotoxic T cells [7]–[10].

By generating FSP-specific CTL clones, we were able to identify two distinct epitopes within MSH3(-1). Since those peptides show no common motif, this formally proofs the induction of (clonally) different T cell responses recognizing the same mutation. Our results show that several MSH3(-1) epitopes can be recognized on tumor cells following natural expression and proteasomal processing. However, the detailed analysis of a high number of CTL clones raised against several FSPs also hints towards a high heterogeneity between clones. Many clones strongly reacted against peptide-loaded target cells (65 of 156). But more than half of those were not able to recognize tumor cells carrying the underlying mutation (35 of 65 did not react). To explain this interesting finding, one may speculate, that T cells of lower avidity fail to efficiently lyse tumor target cells presenting endogenously generated FSP, whereas T cells of high avidity can react against targets displaying low peptide levels.

Furthermore, most of the remaining clones recognizing HCT116 (30) failed to lyse Colo60H (only 5 reacted). Several reasons may explain this finding. Colo60H is generally a poorer target than HCT116 [9 and own unpublished data]; this may for example be contributable to a higher intrinsic resistance towards CTLs. Moreover, surface expression levels of HLA-A2 may differ; but in FACS-analysis we found very similar HLA-A2 expression of the two CRC cell lines (data not shown). Finally, differences in the levels of either MSH3(-1) protein expression or of (peptide)-antigen presentation between HCT116 and Colo60H might be a plausible explanation for this observation. This is an interesting question which shall be addressed in future studies.

In summary, the data presented here suggest that the MSH3(-1) frameshift constitutes – from an immunological point of view – one of the major molecular alterations that take place in MSI^+^ tumors.

However, our data are to some extent in conflict to the findings of a pioneer work performed by Williams and colleagues [21]. They very recently analyzed the sensitivity of MSI-induced frameshift mutations towards nonsense mediated RNA decay and found MSH3(-1) to be decay-sensitive [21]. This finding has two major aspects. First, when MSH3(-1) mRNA is degraded very fast, our observed sensitivity of endogenously MSH3(-1) expressing tumor cells towards specific T cells implies that the described epitopes must be very immunogenic and the raised T cells must be of high avidity. Contrary to that it would secondly raise concerns about the potential of MSH3(-1) protein to be cross-presented by professional antigen-presenting cells and thus would substantially limit it's usefulness as MSI-specific tumor antigen. We here want to bring forward several arguments in favour of the highly immunogenic character of MSH3(-1). A weaker argument would be that all the T cell epitopes derived from cMS that have been described so far [7]–[12] are predicted to be sensitive for nonsense-mediated RNA decay [21]. All of these epitopes were found to be naturally presented and specific T cells could efficiently kill tumor cells endogenously expressing the underlying mutation. Then again, both T cell as well as antibody responses specific for cMSI-induced frameshift antigens have been observed in patients as well as healthy HNPCC mutation carriers [13], [22].

A major obstacle of immunotherapy as a whole is the obvious fact, that a tumor mass – a large and heterogeneous ensemble of genetically unstable cells – uses different mechanisms that will possibly allow at least some tumor cells to survive the highly specific immune attack that can be induced by a single T-cell epitope. In this regard, strategies based on the combination of multi-epitope immunotherapeutic approaches with standard (chemo) therapies may finally be clinically most effective.
